# Supplementary material for: Uncovering microbiomes of the rice phyllosphere using long-read metagenomic sequencing
Source: Commun Biol. 2024 Mar 27;7:357. doi: 10.1038/s42003-024-05998-w (PMC10973392; doi:10.1038/s42003-024-05998-w)
Supplement: Supplementary file 2 — Supplementary Figs. [file 42003_2024_5998_MOESM2_ESM.pdf]

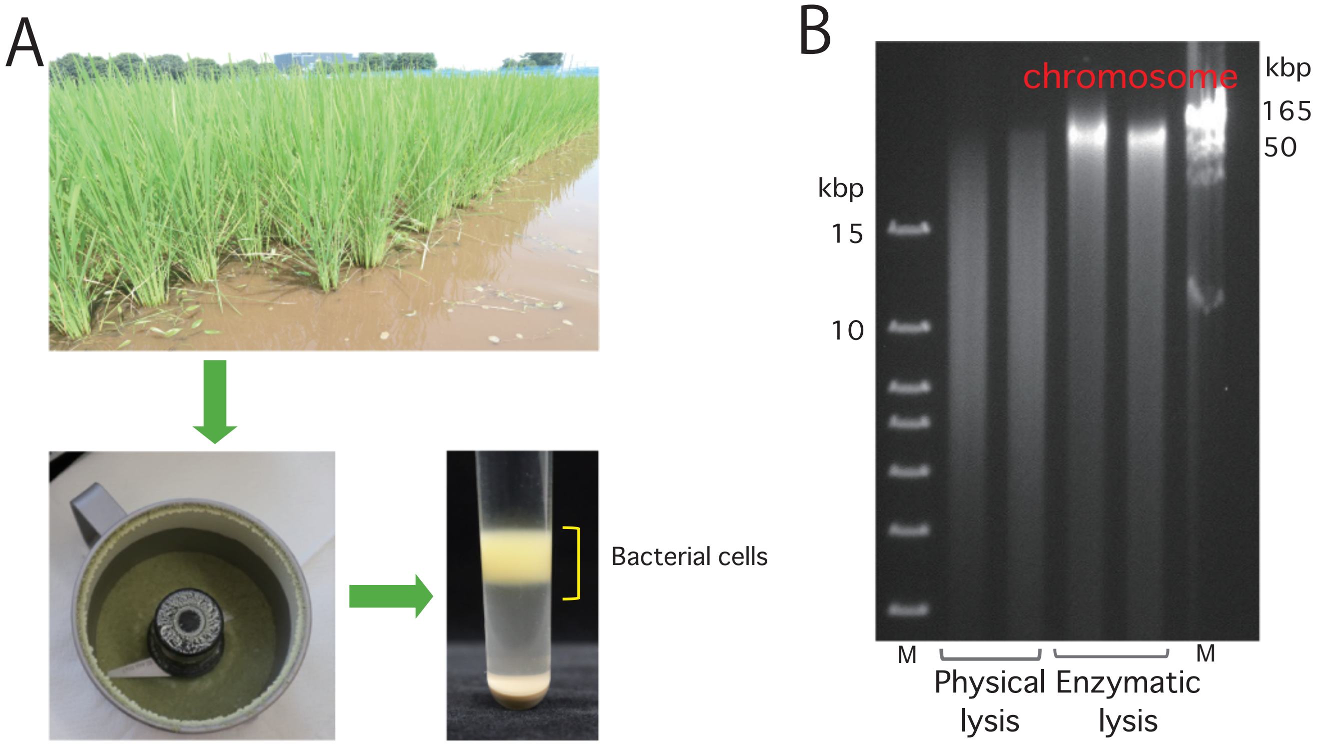

Supplementary Figure 1. Preparation of genomic DNA from the rice-microbiome for long-read metagenomic sequencing.

(A) Rice plants were sampled from experimental field and ground with dry ice. Bacterial cells were purified from aerial parts of rice plants using cell density centrifugation. (B) Genomic DNA was extracted from the purified microbiome using physical and enzymatic lysis. The presence of chromosomal DNA was confirmed using Pulse-field gel electrophoresis. M; marker

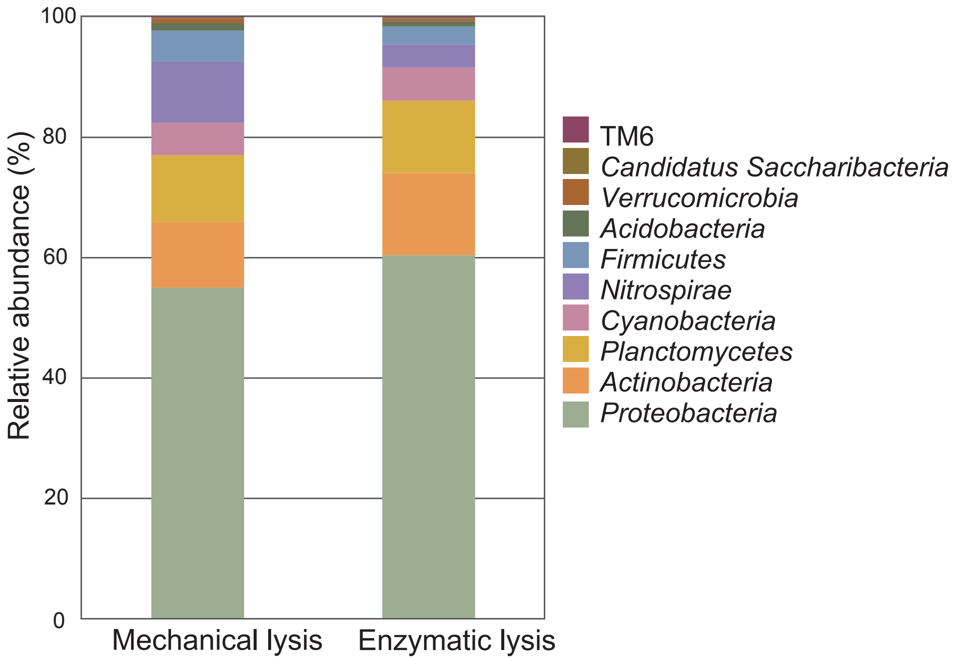

Supplementary Figure 2. Comparison of the relative abundance of 16S rRNA genes extracted with mechanical lysis and enzymatic lysis.

Each color represents the relative abundance of 16S rRNA genes at the corresponding taxonomic rank.

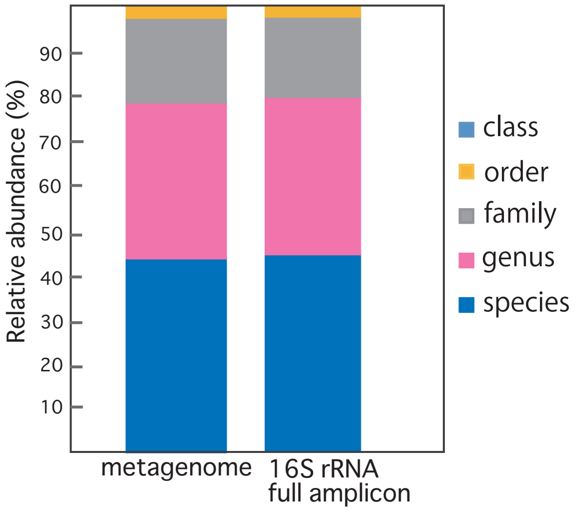

Supplementary Figure 3. Comparison of the relative abundance of 16S rRNA genes at different taxonomical ranks in the metagenome and 16S rRNA full-length amplicon sequences. Each color represents the relative abundance of 16S rRNA genes at the corresponding taxonomic rank.

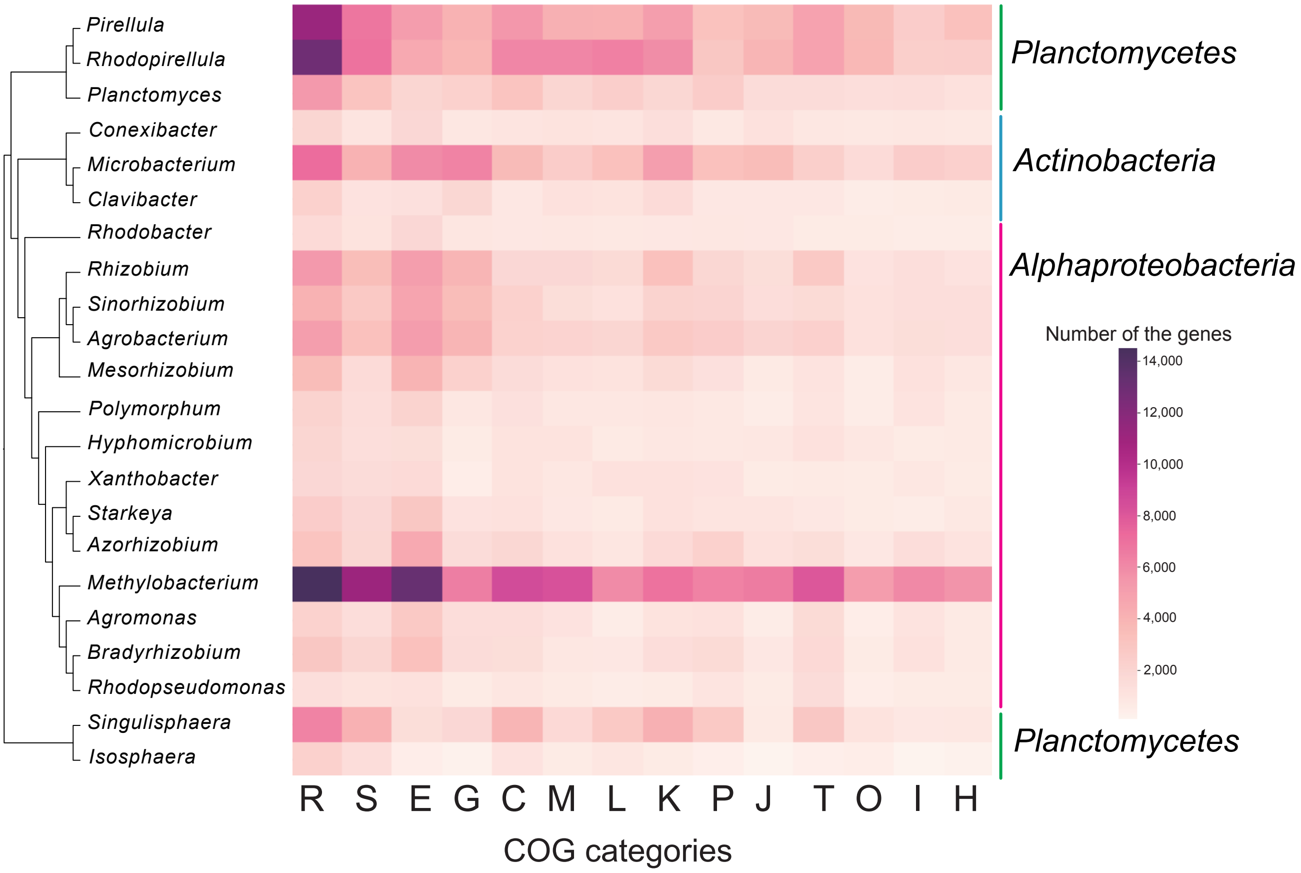

Supplementary Figure 4. Gene categorization using the COG database.
The number of genes categorized in each group is shown in the heatmap.

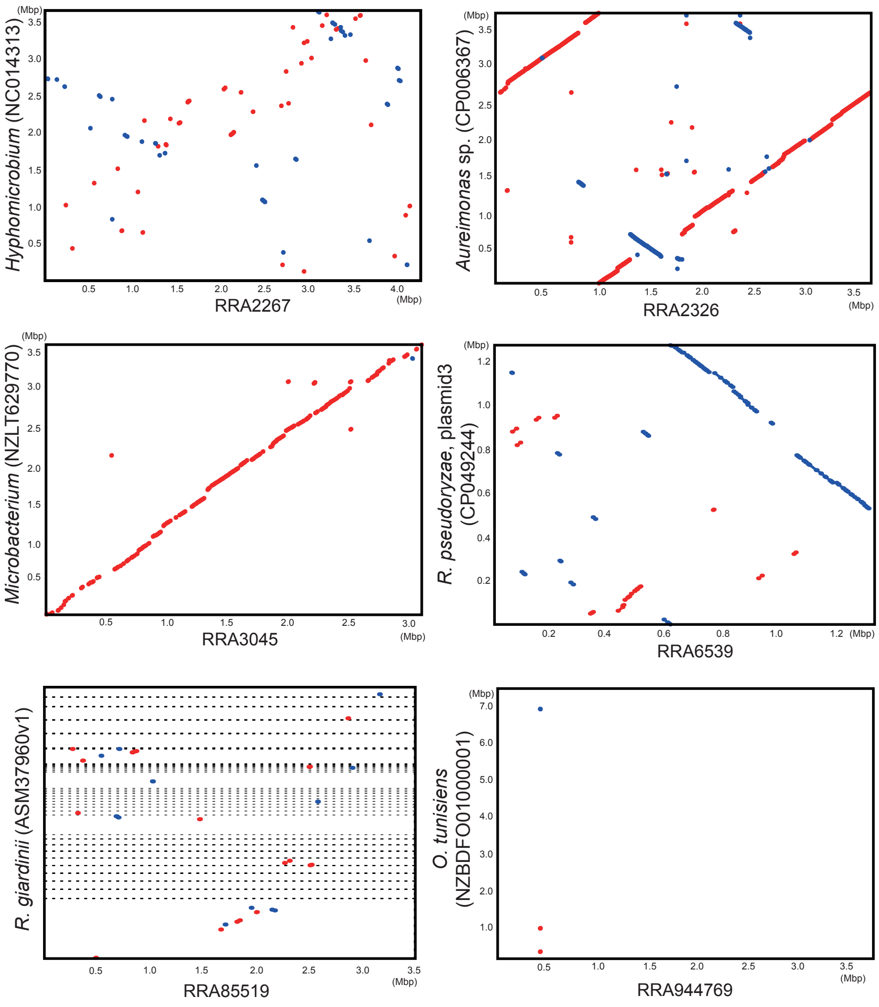

Supplementary Figure 5. Alignment of whole genomic sequences between the six circular contigs and the closest bacterial genome. The genome of the reference strain, *R. giardinii*, was determined by whole genome sequencing (WGS). The bold dotted line of the horizontal axis represents each contig of *R. giardinii*.

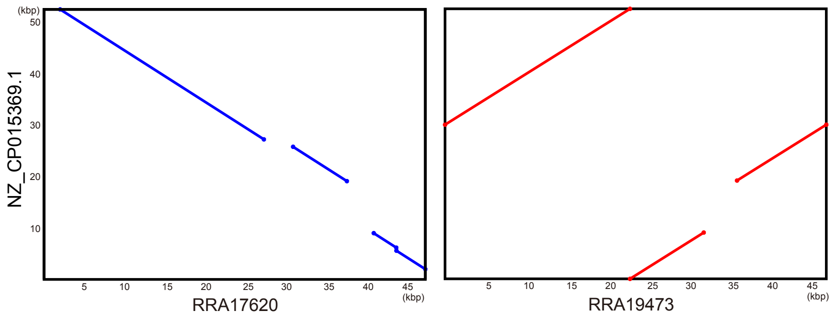

Supplementary Figure 6. Comparison of the plasmid sequence. Comparison of the whole genomic sequences of RRA17620 and RRA19473 to the plasmid of *Methylobacterium phyllosphaerae* strain CBMB27 (NZ_CP015369.1).

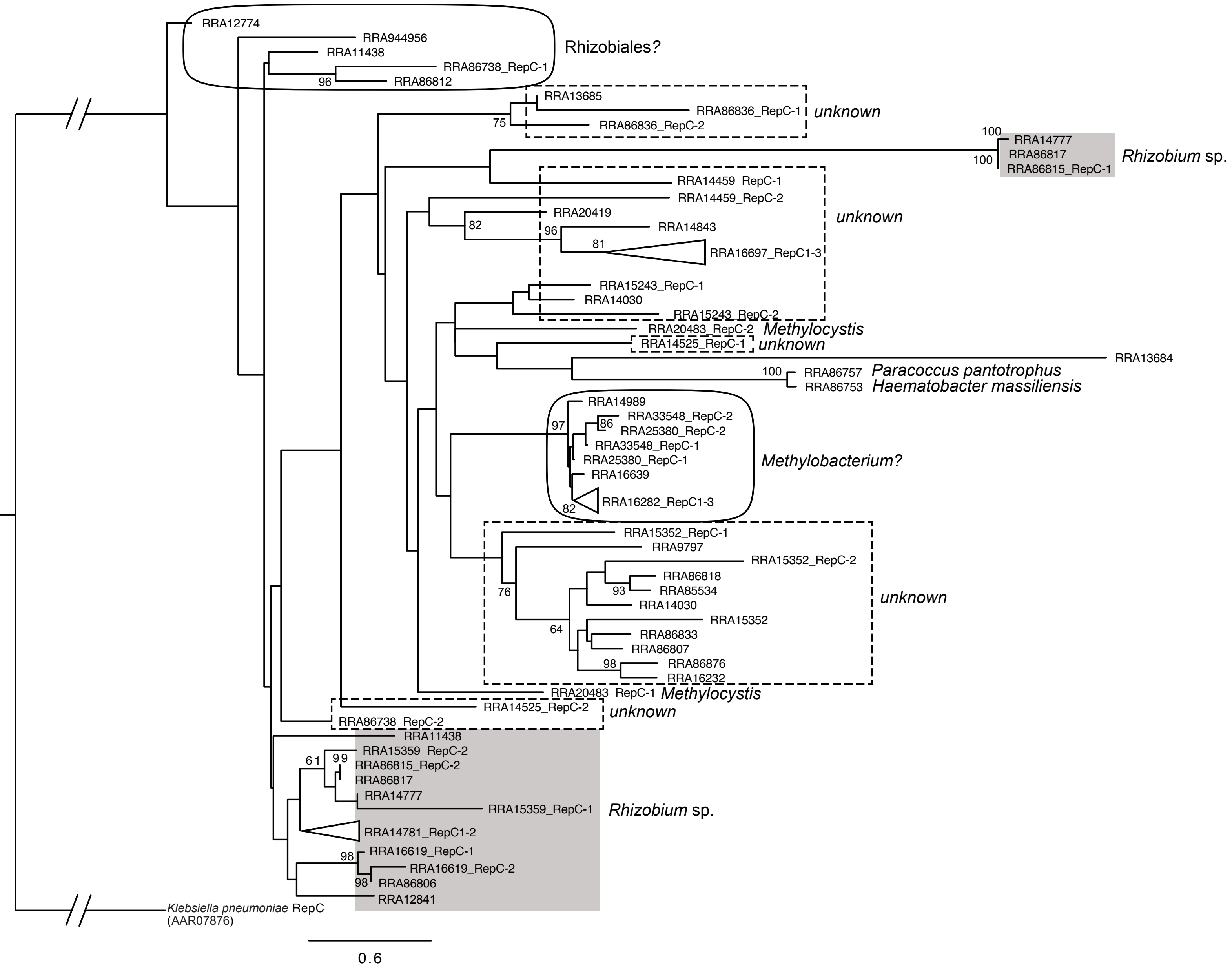

Supplementary Figure 7. Phylogenetic tree of RepC in small circular contigs (< 1Mbp). The 61 of RepC on 39 contigs were used to construct the phylogenetic tree. The accession number indicates the representative RepC in each cluster. The taxonomy of RepC that are independently clustered with known RepC are defined as unknown. The RepC of *Klebsiella pneumoniae* (accession number: AAR07876) was used as the outgroup.

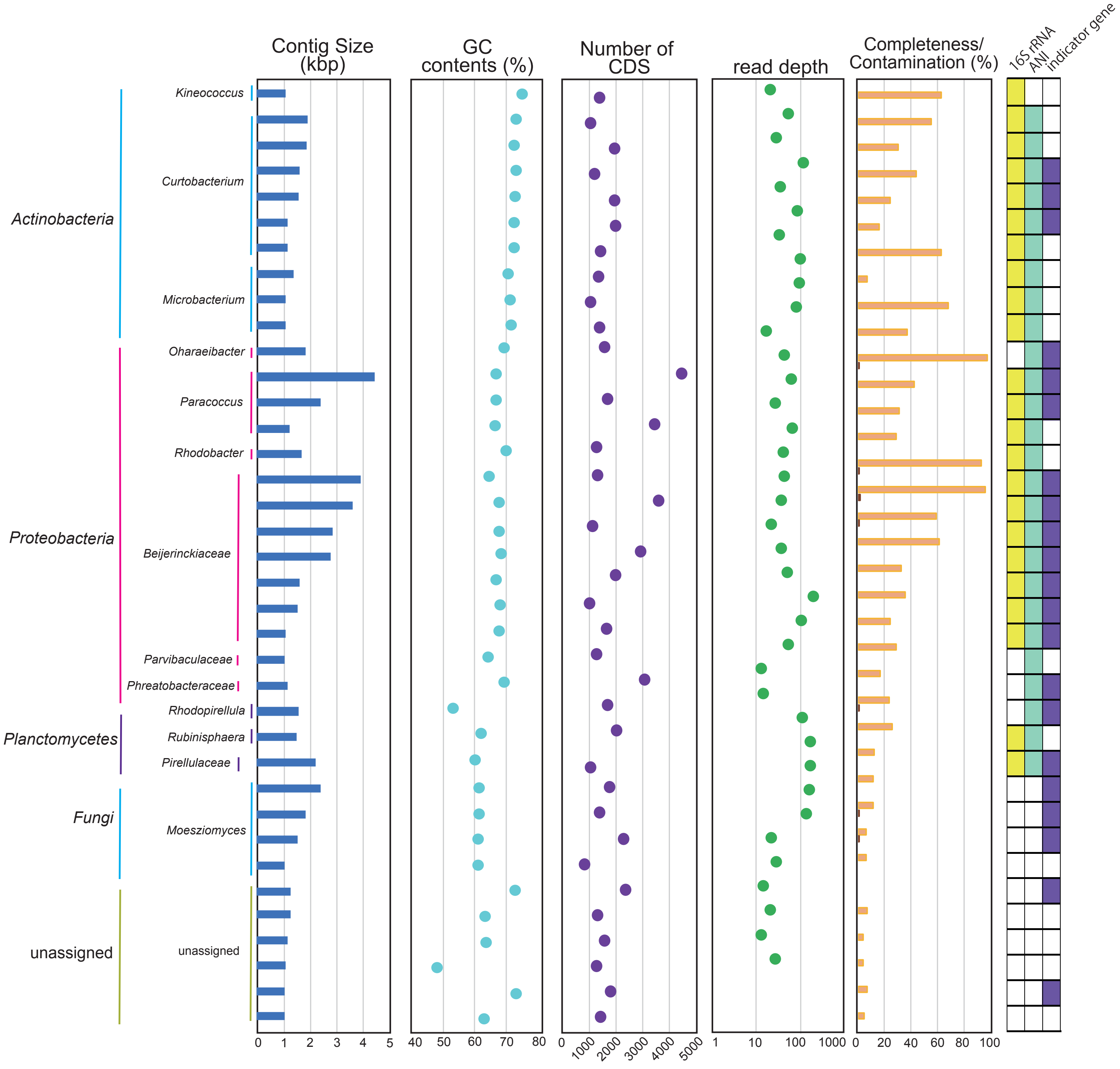

Supplementary Figure 8. Characteristics of large contigs (n=37).
The taxonomic assignment of each contig was determined based on 16S rRNA genes, ANI, and gene similarity searches. Contigs carrying 16S rRNA genes are shown in yellow blocks. Contigs classified by ANI are shown in green blocks. Contigs carrying dnaA and mini-chromosome maintenance genes are shown in purple and pink blocks, respectively. The blue and black bars in the contig size column represent chromosomes and unclassified (neither chromosome nor plasmid), respectively. The completeness (yellow) and contamination (orange) were shown.

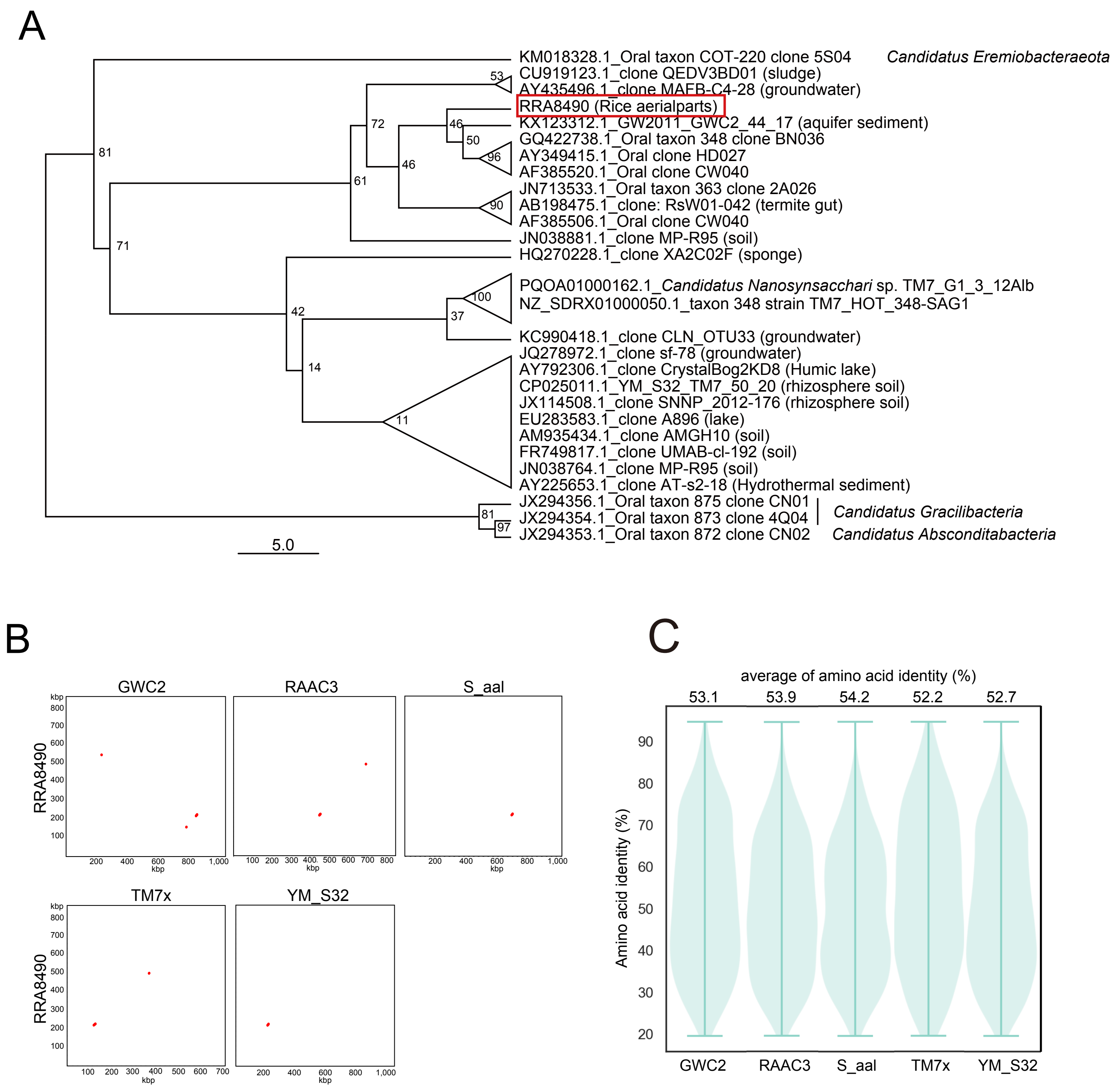

Supplementary Figure 9. Comparison of RRA8490 and other strains in *Candidatus Sacchribacteria.*
(A) a phylogenetic tree using 16S rRNA genes of strains in *Candidatus Sacchribacteria*. (B) Comparison of whole genomic sequences between RRA8490 and five strains. Whole genomic sequences were compared using nucmer, showing that RRA8490 is not similar to the others. (C) amino acid identity between RRA8490 and the five strains in *Candidatus Sacchribacteria*. The average amino acid identity was calculated using the AAI calculator in Kostas lab with default parameters (http://enve-omics.ce.gatech.edu/aai/).
