# Supplementary material for: Uncovering microbiomes of the rice phyllosphere using long-read metagenomic sequencing
Source: Commun Biol. 2024 Mar 27;7:357. doi: 10.1038/s42003-024-05998-w (PMC10973392; doi:10.1038/s42003-024-05998-w)
Supplement: Supplementary file 3 — Description of Additional Supplementary Files [file 42003_2024_5998_MOESM3_ESM.pdf]

Description of Additional Supplementary Files

File name: Supplementary Data
Description: The source data behind the Figures and tables.
