# Supplementary material for: Uncovering microbiomes of the rice phyllosphere using long-read metagenomic sequencing
Source: Commun Biol. 2024 Mar 27;7:357. doi: 10.1038/s42003-024-05998-w (PMC10973392; doi:10.1038/s42003-024-05998-w)
Supplement: Supplementary file 5 — Reporting Summary [file 42003_2024_5998_MOESM5_ESM.pdf]

nature portfolio

Corresponding author(s): Ken Shirasu

Last updated by author(s): Feb 9, 2024

Reporting Summary

Nature Portfolio wishes to improve the reproducibility of the work that we publish. This form provides structure for consistency and transparency in reporting. For further information on Nature Portfolio policies, see our Editorial Policies and the Editorial Policy Checklist.

Statistics

For all statistical analyses, confirm that the following items are present in the figure legend, table legend, main text, or Methods section.

n/a	Confirmed	
☐	☒	The exact sample size (*n*) for each experimental group/condition, given as a discrete number and unit of measurement
☐	☒	A statement on whether measurements were taken from distinct samples or whether the same sample was measured repeatedly
☒	☐	The statistical test(s) used AND whether they are one- or two-sided<br>*Only common tests should be described solely by name; describe more complex techniques in the Methods section.*
☒	☐	A description of all covariates tested
☒	☐	A description of any assumptions or corrections, such as tests of normality and adjustment for multiple comparisons
☒	☐	A full description of the statistical parameters including central tendency (e.g. means) or other basic estimates (e.g. regression coefficient) AND variation (e.g. standard deviation) or associated estimates of uncertainty (e.g. confidence intervals)
☒	☐	For null hypothesis testing, the test statistic (e.g. *F*, *t*, *r*) with confidence intervals, effect sizes, degrees of freedom and *P* value noted<br>*Give P values as exact values whenever suitable.*
☒	☐	For Bayesian analysis, information on the choice of priors and Markov chain Monte Carlo settings
☒	☐	For hierarchical and complex designs, identification of the appropriate level for tests and full reporting of outcomes
☒	☐	Estimates of effect sizes (e.g. Cohen's *d*, Pearson's *r*), indicating how they were calculated

Our web collection on statistics for biologists contains articles on many of the points above.

Software and code

Policy information about availability of computer code

Data collection	PacBio Sequel II
Data analysis	Canu v 1.8, seqkit v0.11.0, pbmm2 v 1.2.1, minimap2 v 2.14, IGV browser v 2.8.2, Quast v 5.0.2, PROKKA v 1.14.6, Interproscan v 5.46-81.0, Kofamscan v1.3.0, Augustus v. 3.4.0, CD-HIT v 4.8.1, SMRTLink v 8.0.0, MAFFT v7.475, RaxML v8.2.12, FigTree v 1.4.4, GTDB-tk v 1.1.1, BLASTN, BLASTP, checkM v1.1.2, nucmer v4.0.0beta2, Plasflow v1.1, MOB-suite v3.0.0, Virsorter2 v2.2.2, CheckV v0.7.0, gggenes (R v3.6.0), BlastKOALA (https://www.kegg.jp/blastkoala/), AAI calculator (http://enve-omics.ce.gatech.edu/aai/)

For manuscripts utilizing custom algorithms or software that are central to the research but not yet described in published literature, software must be made available to editors and reviewers. We strongly encourage code deposition in a community repository (e.g. GitHub). See the Nature Portfolio guidelines for submitting code & software for further information.

Data

Policy information about availability of data

All manuscripts must include a data availability statement. This statement should provide the following information, where applicable:

- Accession codes, unique identifiers, or web links for publicly available datasets
- A description of any restrictions on data availability
- For clinical datasets or third party data, please ensure that the statement adheres to our policy

The data is available in NCBI database. The accession number is PRJNA929667 (BioProject), SAMN32580422 (BioSample), SRX19223815 and SRX19223816 (SRA).

Human research participants

Policy information about studies involving human research participants and Sex and Gender in Research.

Reporting on sex and gender	N/A
Population characteristics	N/A
Recruitment	N/A
Ethics oversight	N/A

Note that full information on the approval of the study protocol must also be provided in the manuscript.

Field-specific reporting

Please select the one below that is the best fit for your research. If you are not sure, read the appropriate sections before making your selection.

☐ Life sciences ☐ Behavioural & social sciences ☒ Ecological, evolutionary & environmental sciences

For a reference copy of the document with all sections, see nature.com/documents/nr-reporting-summary-flat.pdf

Ecological, evolutionary & environmental sciences study design

All studies must disclose on these points even when the disclosure is negative.

Study description	a metagenomic analysis using long-read sequences to reveal the genetic makeup of rice leaf microbiome
Research sample	Rice plants (Oryza sativa cultivar 'Koshihikari')
Sampling strategy	Rice plants were collected from an experimental paddy field since we obtain the required amount of gDNA for PacBio Sequel II sequencing. The sample size was estimated by previous reports.
Data collection	The rice roots and aerial parts were separated and stored at -80 °C before use. The aerial parts were grounded, and plant-associated microbes were enriched using a bacterial cell enrichment method. Genomic DNA was extracted from the enriched microbes using enzymatic lysis. The library for sequencing was constructed and sequenced within a week after DNA extraction. SMRTbell libraries for sequencing were constructed according to the manufactural protocol without shearing. The libraries were cut off at 20 kbp using the BluePippin size selection system and sequenced on two SMRT Cells 8M.
Timing and spatial scale	The rice plants were sampled before heading on August 6th, 2018
Data exclusions	No data were excluded
Reproducibility	N/A
Randomization	Rice plants were randomly sampled from the paddy field on the same day
Blinding	No blinding was performed

Did the study involve field work? ☒ Yes ☐ No

Field work, collection and transport

Field conditions	The information is available at the Institute for Sustainable Agro ecosystem Services, Graduate School of Agricultural and Life Sciences, The University of Tokyo when researches inquire (https://www.isas.a.u-tokyo.ac.jp/en/page/about/sample)
Location	An experimental paddy field at the Institute for Sustainable Agro ecosystem Services, Graduate School of Agricultural and Life Sciences, The University of Tokyo (35°74'N, 139°54'E)
Access & import/export	Sampling site access (https://www.isas.a.u-tokyo.ac.jp/en/)
Disturbance	No disturbance

Reporting for specific materials, systems and methods

We require information from authors about some types of materials, experimental systems and methods used in many studies. Here, indicate whether each material, system or method listed is relevant to your study. If you are not sure if a list item applies to your research, read the appropriate section before selecting a response.

Materials & experimental systems

n/a	Involved in the study
☒	☐ Antibodies
☒	☐ Eukaryotic cell lines
☒	☐ Palaeontology and archaeology
☒	☐ Animals and other organisms
☒	☐ Clinical data
☒	☐ Dual use research of concern

Methods

n/a	Involved in the study
☒	☐ ChIP-seq
☒	☐ Flow cytometry
☒	☐ MRI-based neuroimaging
